# Detecting Pseudothrombocytopenia in the Era of Artificial Intelligence: Integration of Automated Hematology, Digital Morphology, and Expert Review

**DOI:** 10.7759/cureus.110690

**Published:** 2026-06-11

**Authors:** Enoch Chi Ngai Lim, Nga Chong Lisa Cheng, Chi Eung Danforn Lim

**Affiliations:** 1 Translational Research Department, Specialist Medical Services Group, Earlwood, AUS; 2 Data Science Institute, University of Technology, Sydney, Sydney, AUS; 3 NICM Health Research Instititute, Western Sydney University, Westmead, AUS

**Keywords:** artificial intelligence, automated hematology analyser, edta, platelet clumping, pseudothrombocytopenia

## Abstract

Pseudothrombocytopenia is an artefactual reduction in platelet count that occurs during laboratory testing, most commonly because ethylenediaminetetraacetic acid (EDTA) induces in vitro platelet aggregation. Although clinically benign, it may lead to unnecessary investigations, treatment, transfusion, referral, procedural delay, and patient anxiety when not recognised. Advances in hematology analyzers, including impedance, optical, fluorescence, digital morphology, and artificial intelligence (AI)-assisted technologies, offer opportunities to improve recognition of platelet aggregation and reduce reporting of artefactual thrombocytopenia. This narrative review summarises literature relating to pseudothrombocytopenia, automated platelet counting, digital morphology systems, and AI applications in hematology. Current evidence indicates that analyzer flags and platelet histograms provide useful screening signals but should not replace peripheral blood smear review. Optical and fluorescence platelet channels can improve platelet counting in selected EDTA-dependent samples, while digital morphology systems facilitate documentation of platelet aggregates and support platelet estimation. Emerging AI-assisted workflows are best understood as workflow-support tools that integrate analyzer data, sample timing, channel discordance, digital images, and expert review; they should not be treated as autonomous diagnostic systems. The strongest practical model combines automated platelet channels, digital morphology, AI-supported triage, clear report communication, and expert clinical oversight. Evidence remains heterogeneous, largely platform-specific, and limited by the lack of direct AI-versus-conventional workflow comparisons. Future research should validate integrated systems across diverse laboratory environments and assess clinically relevant outcomes, including diagnostic accuracy, false-positive and false-negative consequences, workflow efficiency, cost, and patient management.

## Introduction and background

Pseudothrombocytopenia is an avoidable laboratory artefact encountered in clinical hematology practice. In affected samples, diagnostic testing may report a spuriously low platelet count because platelets aggregate in vitro rather than because the patient has true thrombocytopenia. ethylenediaminetetraacetic acid (EDTA)-dependent pseudothrombocytopenia is the most frequently described form and is sometimes referred to as a “phantom platelet” problem. If the artefact is not recognised, the apparent thrombocytopenia may prompt unnecessary repeat testing, urgent referral, platelet transfusion, corticosteroid or immunoglobulin treatment, procedural delay, or investigation for disorders that are not present [1].

The biological basis is well described. EDTA chelates calcium and may expose platelet membrane epitopes, particularly on glycoprotein IIb/IIIa. Naturally occurring or acquired antibodies may then bind these epitopes and induce platelet clumping in vitro. The patient is often asymptomatic and may have no bleeding, petechiae, purpura, or other evidence of platelet consumption. Although the prevalence is low in unselected populations, pseudothrombocytopenia is more common among patients investigated for isolated thrombocytopenia, and it may remain clinically silent for long periods if platelet counts are interpreted without attention to the laboratory context [2-4].

Conventional recognition relies on the combination of clinical-laboratory discordance, analyzer warning flags, platelet histograms, and peripheral blood smear review. Impedance platelet counting estimates platelet number from electrical resistance changes as particles pass through an aperture; this method is vulnerable to platelet clumps, giant platelets, fragments, and particle overlap. Optical and fluorescence platelet counting use light scatter and/or fluorescent staining characteristics to better separate platelets from interfering particles and may recover a more reliable count in selected samples. These technologies improve the analytical response to suspected artefact, but abnormal histograms or analyzer flags remain screening signals rather than confirmatory evidence. A smear review remains essential when the numerical result conflicts with the clinical picture or when platelet clumps are suspected [5,6].

Digital morphology systems extend smear review by scanning, storing, and displaying selected smear regions for local or remote assessment. In this context, artificial intelligence (AI)-assisted analysis refers to algorithmic support for image classification, candidate platelet aggregate detection, platelet estimation, reflex-rule prioritisation, and workflow routing. The clinical rationale for applying AI to pseudothrombocytopenia is not that pseudothrombocytopenia requires an autonomous diagnosis. Rather, the unmet need is operational and safety-related: clinically important weak signals are distributed across platelet count, analyzer flags, channel discordance, sample timing, smear quality, prior results, and the final report comment. A well-designed AI-supported workflow can help integrate these signals, prioritise cases for human review, and reduce the risk that a misleading platelet count is released without appropriate qualification.

Digital morphology and AI, therefore, sit between analyzer-based screening and expert validation. The relevant question is not whether AI can replace morphologists, but whether integrated automated systems can help laboratories detect, confirm, document, and communicate pseudothrombocytopenia more consistently while preserving human oversight. This review focuses on that clinically driven question and distinguishes direct pseudothrombocytopenia evidence from adjacent evidence on platelet estimation, digital morphology, and broader AI implementation in laboratory medicine [7-9].

Methods

This article is a narrative review based on a focused literature search rather than a systematic review protocol. The aim was to summarise practical evidence relevant to pseudothrombocytopenia detection and to identify implementation issues for automated hematology, digital morphology, and AI-supported workflows.

PubMed and Google Scholar were searched through May 21, 2026. Representative PubMed search strings included: (“pseudothrombocytopenia” OR “pseudo thrombocytopenia” OR “EDTA-dependent pseudothrombocytopenia” OR “spuriously low platelet count” OR “platelet clumping”) AND (“automated hematology analyzer” OR “platelet count” OR “platelet histogram” OR “platelet clump flag” OR “optical platelet” OR “fluorescence platelet”); and (“digital morphology” OR “digital morphology analyzer” OR “MC-80” OR “DI-60” OR “artificial intelligence” OR “machine learning”) AND (“platelet” OR “platelet estimate” OR “hematology”). Google Scholar searches used the same terms in combination with platform names, including BC-6800, BC-6800Plus, CAL-8000, Sysmex XN, XN-9100, DI-60, and MC-80. Reference lists of relevant reviews and studies were also hand-screened.

Articles were included when they addressed one or more of the following areas: EDTA-dependent pseudothrombocytopenia; spuriously low platelet counts; analyzer flags or histograms; optical or fluorescence platelet counting; digital morphology systems; platelet estimation; or AI-supported hematology workflows. Case reports were included when they illustrated diagnostic or workflow issues. Studies were excluded when they focused only on true thrombocytopenia without a laboratory artefact, platelet function testing, or non-hematology imaging.

Data extraction focused on study design, analyzer platform, platelet counting method, sample size when reported, platelet clump detection, correction of spurious platelet counts, validation comparator, and workflow implications. Findings were grouped into clinical recognition, analyzer-based detection, digital morphology evidence, AI-assisted platelet estimation, integrated workflows, implementation barriers, and future research priorities. Because the objective was narrative synthesis, no protocol registration, formal PRISMA flow diagram, formal risk-of-bias scoring, Grading of Recommendations Assessment, Development, and Evaluation (GRADE) assessment, or publication-bias assessment was performed. To avoid overstating conclusions, Table 2 identifies the design, sample size where available, and whether each evidence source is directly pseudothrombocytopenia-specific, adjacent platelet/digital morphology evidence, or broader workflow evidence. A meta-analysis was not performed because studies used different analyzers, thresholds, sample types, reference methods, and outcome measures. Quantitative findings are therefore reported descriptively rather than pooled statistically.

## Review

Results

Clinical Recognition of Pseudothrombocytopenia

**Figure 1:**
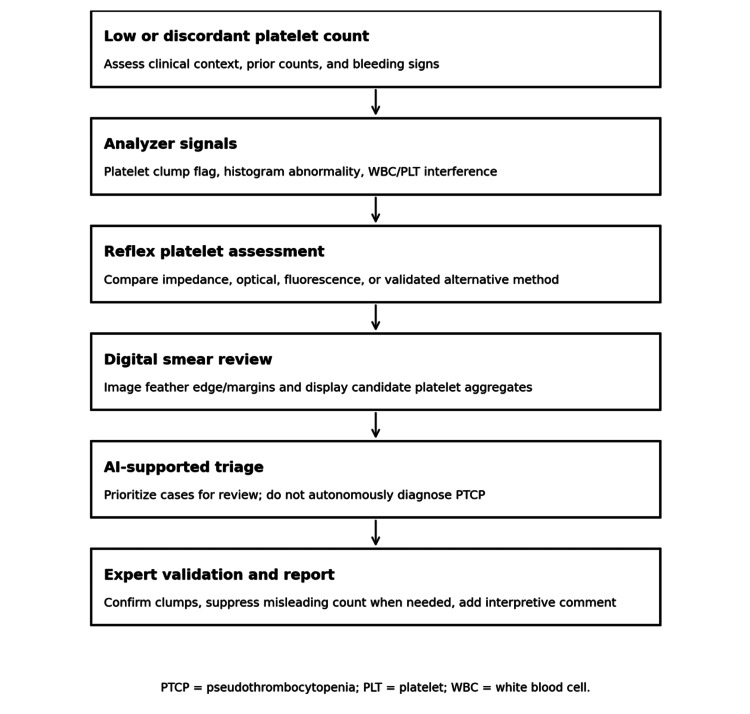
Suggested integrated workflow for suspected pseudothrombocytopenia (PTCP) Note: The workflow is an author-derived synthesis. It illustrates where AI-supported triage may be useful while preserving expert review before a final interpretive report is issued.

Pseudothrombocytopenia detection begins with pattern recognition rather than a single diagnostic test. The key pattern is a low platelet count that conflicts with the patient’s clinical condition or previous results. Analyzer flags, abnormal histograms, platelet clumps on smear, channel discordance, time-dependent count changes, and improved counts with alternative methods all add information. Table 1 summarises practical trigger points for AI-supported recognition. The recommended laboratory actions are labelled as 'evidence-supported' when they are directly grounded in cited pseudothrombocytopenia or digital morphology literature and as author-derived synthesis when they translate the evidence into a proposed local workflow step. These labels are intended to prevent the framework from being interpreted as a validated international guideline. Figure 1 illustrates a proposed integrated workflow for suspected pseudothrombocytopenia, beginning with a low or discordant platelet count, followed by analyzer flag review, alternative platelet channel assessment, digital smear evaluation, AI-supported triage, expert validation, and final report communication.

**Table 1:** Clinical and laboratory triggers for AI-supported pseudothrombocytopenia detection Note: “Evidence-supported” refers to actions directly supported by cited pseudothrombocytopenia, platelet-counting, or digital morphology literature. “Author-derived synthesis” refers to practical workflow recommendations inferred from that evidence and requiring local validation.

Trigger point	Typical finding	Automated or AI-supported contribution	Recommended laboratory action and evidence basis	Key sources
Clinical-laboratory discordance	Low platelet count without bleeding, petechiae, purpura, or other thrombocytopenia signs.	Rule-based alert can compare platelet count with prior counts and available clinical comments.	Evidence-supported: review smear before urgent treatment or referral when the platelet result is clinically discordant.	[1-3]
Analyzer platelet clump flag	Platelet clump flag, abnormal platelet histogram, or unusual WBC/platelet distribution.	Analyzer can trigger reflex smear imaging, alternate platelet channel testing, or supervisory review.	Evidence-supported: confirm platelet clumps by microscopy before accepting a critically low count.	[2,5]
Channel discordance	Low impedance platelet count with higher optical or fluorescence count.	Multi-channel comparison can identify possible count interference.	Evidence-supported with local validation: report the validated channel with an interpretive comment when appropriate.	[6,10]
Time-dependent platelet fall	Platelet count decreases after ethylenediaminetetraacetic acid sample storage or transport delay.	Laboratory information system can track time from collection to analysis and trigger repeat testing.	Author-derived synthesis from pseudothrombocytopenia evidence: repeat promptly or use a validated alternative method when time-dependent clumping is suspected.	[2,10]
Persistent low count in the alternative tube	Citrate or heparin count remains low because of multi-anticoagulant clumping or inadequate dissociation.	The algorithm can prevent false reassurance from a single alternative tube result.	Evidence-supported caution plus author-derived workflow: use smear review, rapid analysis, or specialised platelet method rather than relying solely on one alternative anticoagulant.	[2,3,10]
Digital smear evidence	Platelet clumps at the feather edge, smear margins, or low-power fields.	Digital morphology can display candidate clump images and support remote or second review.	Evidence-supported: add an interpretive comment and suppress or qualify a misleading platelet count when clumps compromise reliability.	[7,8,11]

Analyzer-Based Detection and Channel Discordance

Automated hematology analyzers have progressed from simple count generation to multi-signal interpretation. Impedance platelet counting remains vulnerable to platelet clumps, giant platelets, and particle overlap. Optical and fluorescence channels can reduce some of these errors by leveraging additional scatter and fluorescence characteristics. Bao and colleagues tested EDTA-dependent pseudothrombocytopenia samples on BC-6800 and XE-2100 systems. The BC-6800 optical fluorescence method produced platelet counts comparable with citrate samples in many cases; 22 of 23 samples showed more than 80% platelet dissociation, with an average dissociation rate of 93% [6]. This supports fluorescence platelet channels as a useful reflex step, but the small sample size, platform specificity, and continued need for smear correlation limit generalisability.

Time, anticoagulant type, and analyzer channel also influence platelet recovery. Zhang and colleagues studied 43 EDTA-dependent pseudothrombocytopenia samples using Sysmex and Mindray analyzers. Platelet counts varied according to storage time, anticoagulant, and detection channel. The XN-9100 fluorescence platelet channel showed better platelet dissociation in some cases, while Mindray optical counting also provided a useful retesting option [10]. These results support time-sensitive and channel-aware reflex pathways, but they also indicate that no universal tube, channel, or delay threshold can be assumed across laboratories.

Digital Morphology Evidence

Digital morphology systems expand the response to suspected pseudothrombocytopenia by documenting platelet clumps and providing images for expert review. They may help identify aggregates at the feather edge or smear margins, where clumps may be missed during rapid manual review. So and colleagues assessed an MC-80 digital image analyzer integrated with a BC-6800Plus hematology analyzer in suspected EDTA-induced pseudothrombocytopenia. Conventional analyzer flagging showed modest sensitivity to platelet clumps, sensitivity improved with MC-80 digital imaging, and the PLT-Pro approach achieved complete sensitivity in the study setting [11]. These findings support digital morphology as an adjunct to smear review, while also underlining dependence on smear quality, platform-specific rules, and local validation.

AI-Assisted Platelet Estimation and Integrated Workflows

AI-assisted platelet estimation is emerging as an adjunct when a numeric platelet count is unreliable or requires verification. Guo and colleagues evaluated AI-assisted morphological analysis for platelet count estimation using the MC-80 digital morphology analyzer in 977 samples across low, medium, and high platelet ranges, with a subset compared with immunologic platelet counting. AI-supported morphology improved platelet transfusion decision support, especially in low platelet samples [12]. Tantanate evaluated platelet estimation factors for the Sysmex DI-60 digital morphology analyzer and showed that adjusted estimation factors can improve platelet count estimation [13]. Üstündağ and colleagues assessed MC-80 estimated platelet counts as an adjunct to automated hematology analyzers, including samples with aggregation or platelet clump flags [14]. These studies are relevant to platelet verification, but they are not all specific to EDTA-dependent pseudothrombocytopenia; therefore, they provide adjacent evidence rather than definitive proof of AI superiority in pseudothrombocytopenia detection.

Integrated platelet workflows offer the most clinically relevant model because they combine multiple weak signals. Giovannelli and colleagues validated an intelligent automated workflow using Mindray BC-6800Plus platelet impedance, platelet optical counting, automated smear preparation, MC-80 morphology, and immunologic platelet comparison in 1,208 samples. They reported a strong correlation with immunologic platelet methods in abnormal samples and more than 99% workflow accuracy [15]. Guy and colleagues tested an AI- and morphology-driven workflow that integrated four platelet enumeration technologies in 2,474 routine EDTA samples; the workflow produced accurate platelet evaluation, including cases affected by platelet clumps [16]. These studies show feasibility and high within-study performance, but their estimates should be interpreted cautiously because external validation, spectrum effects, local calibration, and the effect on clinical outcomes remain incompletely established. Table 2 maps the key evidence base for these technologies.

**Table 2:** Evidence map of automated and AI-assisted platelet technologies relevant to pseudothrombocytopenia (PTCP) Note: Evidence directness is descriptive and is not a formal level-of-evidence grade. No formal risk-of-bias scoring was performed because this was a narrative review.

Technology or workflow	Study design, sample size, and directness of evidence	Main finding	Practical strength	Main limitation or caution
Analyzer flags and histograms	Case-based and review evidence; sample size not applicable; direct PTCP recognition evidence.	Flags and abnormal histograms can suggest platelet clumping.	Fast first-line trigger for smear review.	Insufficient sensitivity to exclude clumps; negative flag does not rule out PTCP [2,5].
Optical fluorescence platelet counting	Analyzer evaluation in ethylenediaminetetraacetic acid (EDTA)-dependent PTCP; n=23; direct PTCP evidence.	Many spurious low counts were corrected without a second sample; 22/23 samples showed >80% dissociation.	Useful reflex platelet channel in selected samples.	Small sample size and platform-specific performance; smear correlation remains needed [6].
Time- and channel-based retesting	Multi-analyzer study of EDTA-dependent PTCP; n=43; direct PTCP evidence.	Platelet count depends on time, anticoagulant, and channel.	Supports time-sensitive reflex algorithms.	No universal best tube, channel, or delay threshold across laboratories [10].
BC-6800Plus plus MC-80 digital imaging	Suspected EDTA-induced PTCP workflow study; direct PTCP workflow evidence.	Integration improved platelet clump detection sensitivity compared with conventional flagging.	Links count data with visual evidence for review.	Requires smear quality control and local platform validation [11].
AI-assisted MC-80 platelet estimation	Morphological platelet estimation study; n=977; adjacent platelet-estimation evidence.	AI morphology supported platelet estimation and transfusion decision support, especially at low platelet counts.	Supports verification when the automated count is uncertain.	Not specific to the EDTA artefact alone; comparator and local thresholds matter [12].
DI-60 platelet estimate factor	Digital morphology platelet estimation study; adjacent platelet estimation evidence.	Adjusted estimate factors improved platelet count estimation.	May standardise smear-based platelet estimation.	Estimate factors may require local calibration and are not directly PTCP-specific [13].
MC-80 estimated platelet count adjunct	Digital morphology adjunct study, including aggregation/clump flags; adjacent-to-direct platelet workflow evidence.	Estimated platelet counts may support the interpretation of abnormal automated results.	Helpful when analyzer count requires verification.	Performance may vary by smear quality, platelet range, and local review practice [14].
Intelligent automated platelet workflow	Automated workflow validation; n=1,208; broad abnormal platelet workflow evidence.	High automated validation accuracy and strong correlation with the immunologic platelet method were reported.	Reduces manual review burden and supports reflex testing.	External validity, local calibration, and clinical outcome effects remain uncertain [15].
Four-technology AI morphology workflow	Routine EDTA workflow study; n=2,474; broad platelet workflow evidence, including clumped samples.	Integrated workflow produced accurate platelet evaluation, including clumped samples.	Demonstrates feasibility in routine workflow.	Evidence demonstrates feasibility more than definitive superiority over conventional expert review [16].

Discussion

Clinical Rationale for AI-Supported Pseudothrombocytopenia Workflows

The central finding of this review is that AI is most defensible when it supports laboratory workflow rather than when it is presented as a stand-alone diagnostic tool. Pseudothrombocytopenia cannot be resolved by one platelet count because the artefact is influenced by antibody-dependent aggregation, anticoagulant exposure, sample age, analyzer channel, smear quality, and the reporting process. AI can be clinically useful by connecting these weak signals and converting them into a timely review pathway. This framing addresses the actual unmet need: preventing the release of a misleading low platelet count while avoiding unnecessary manual review of every low platelet result.

In a busy laboratory, a platelet clump flag may be overlooked, a platelet histogram may not be reviewed, or a low platelet count may be reported before the discordance is appreciated. An AI-supported rule can combine platelet count, clump flag, histogram pattern, channel discordance, time since collection, prior platelet values, and smear image availability. Such a system should not diagnose the patient. It should identify specimens that require reflex testing, digital smear review, or expert validation before the result is released.

Digital Morphology, Reporting, and Human Oversight

Digital morphology can provide visual documentation of platelet aggregates and can support remote review. It may also reduce variability by standardising image capture and highlighting regions likely to contain clumps. However, rare platelet morphologies, giant platelets, platelet satellitism, atypical aggregates, poor smear quality, stain variability, and edge-of-smear artefacts may reduce algorithmic reliability. Expert morphologist oversight remains important for discordant, critical, or unresolved cases, especially when the digital system identifies candidate clumps, but the reportability of the platelet count remains uncertain [7,8].

The reporting step is equally important. Pseudothrombocytopenia is not only an analytical issue but also a communication issue. A useful report should state when platelet clumping was observed, indicate that the automated count may be falsely low, and describe whether an alternative channel, smear estimate, repeat sample, or specialist review is recommended. If a reliable numerical count cannot be obtained, the report should explain the limitation rather than release a single unqualified platelet value. This approach reduces the risk of unnecessary transfusion, inappropriate treatment, and clinical escalation based on an artefact [1-3].

Failure Modes, Bias, and Governance

False-negative and false-positive AI outputs have different consequences. A false-negative output may allow an artefactually low platelet count to be reported as true thrombocytopenia, potentially causing unnecessary treatment or delay. A false-positive output may increase smear review workload, prolong turnaround time, and create unnecessary clinician concern. Algorithmic bias may also arise if training data are dominated by a single analyzer platform, stain protocol, population, tube type, transport condition, or smear preparation method. Laboratories adopting AI-supported platelet workflows should therefore validate performance locally, monitor override rates and missed-clump events, and revalidate after software, hardware, staining, transport, or workflow changes [9,17,18].

International recommendations for digital morphology emphasise the need for validation, quality control, and expert review, but pseudothrombocytopenia -specific AI recommendations remain limited. Consequently, frameworks such as Table 3 should be interpreted as implementation guidance derived from the current literature and laboratory reasoning, not as validated consensus recommendations. They are intended to help laboratories structure local validation and audit rather than replace professional judgement.

**Table 3:** Implementation framework for AI-assisted pseudothrombocytopenia detection Note: This framework is author-derived and requires local validation. It is not intended to represent formal guideline recommendations or a substitute for laboratory accreditation requirements.

Workflow domain	Minimum requirement	Useful performance measure	Risk if omitted	Supporting evidence
Pre-analytical control	Record tube type, collection time, analysis time, and transport conditions.	Median time to platelet analysis; proportion analysed within local target.	Time-dependent platelet clumping may be missed.	[2,10]
Analyzer rule set	Define reflex criteria using platelet count, clump flag, histogram pattern, and channel discordance.	False-negative clump rate; reflex smear rate; proportion of low counts reviewed.	Low counts may be released without review.	[5,6,11]
Alternative platelet channel	Validate optical or fluorescence platelet count against local reference or expert-reviewed method.	Agreement with smear review or immunologic platelet count.	An incorrect channel may be trusted without local evidence.	[6,10,15]
Digital morphology protocol	Ensure imaging includes regions where platelet clumps are likely to appear.	Clump detection sensitivity, rejected slide rate, and second-review discrepancy rate.	Clumps at the smear edge may be missed.	[7,8,11]
AI-assisted platelet estimation	Calibrate estimate factors for the local platform, smear method, and platelet range.	Agreement across low, medium, and high platelet strata; reviewer override rate.	The estimate may be biased in thrombocytopenic or clumped samples.	[12-14]
Report communication	Use clear interpretive comments when platelet clumps affect count reliability.	Corrected report frequency, clinician callback frequency, and repeat sample rate.	False thrombocytopenia may trigger treatment or referral.	[1-3]
Human oversight	Require expert review for discordant, critical, or unresolved samples.	Reviewer override rate, discrepancy audit rate, and false-negative event review.	Automation bias may suppress expert judgement.	[7-9]
Model governance	Revalidate after software, stain, hardware, tube, transport, or workflow changes.	Drift metrics, external quality assessment results, and local audit outcomes.	Performance may decline without detection.	[17,18]

Limitations and Future Research Direction

Limitations of the evidence base and narrative review method: The principal limitation of the current evidence base is the lack of platform-independent validation. Most studies are single-centre evaluations of specific analyzers, digital morphology systems, or reflex workflows. This limitation is partly expected because platelet counting technologies are instrument-dependent, but it means that published thresholds and performance estimates should not be adopted without local verification. Pseudothrombocytopenia is also biologically heterogeneous. Although EDTA is the commonest trigger, platelet clumping may occur in citrate, heparin, or multiple anticoagulants. A workflow that stops after a citrate repeat may therefore misclassify some cases [2,3,10].

This manuscript should also be interpreted within the inherent limits of a narrative review. Although a focused search and representative search strings were provided, the review was not designed to identify every eligible record, generate a reproducible PRISMA record count, or formally grade the certainty of evidence. Study selection and interpretation may be influenced by author judgment, database coverage, language, publication availability, and citation chaining. Publication bias is also possible: reports demonstrating high accuracy, successful analyzer reflex testing, or favourable digital morphology and AI implementation are more likely to be published than negative, inconclusive, or failed local validations. Manufacturer- or platform-specific validation studies may further overrepresent performance under optimized conditions. Accordingly, the conclusions should be read as a clinically oriented synthesis rather than as a pooled or graded estimate of diagnostic accuracy.

Future Research Priorities

The direct evidence comparing AI-assisted and conventional pseudothrombocytopenia workflows remains limited. Many studies report correlation, sensitivity, validation accuracy, or workflow efficiency, but few assess whether AI reduces unnecessary platelet transfusion, hematology referral, bone marrow biopsy, surgical delay, emergency escalation, or patient anxiety. Future studies should report false-positive and false-negative consequences, turnaround time, workload, cost, interobserver variability, clinician interpretation of comments, and patient-centred outcomes. External validation across laboratories with different analyzers, tubes, transport conditions, smear preparation methods, patient populations, and staffing models is especially important.

The next phase of pseudothrombocytopenia research should examine not only whether AI-supported systems detect platelet clumps accurately, but also how they influence laboratory decisions, reporting practice, clinician behaviour, and patient outcomes. In designing such studies, broader clinical AI concepts can be translated into laboratory-specific evaluation questions. Work on medical synthesis emphasises the integration of multiple information streams rather than reliance on a single automated output [19], which mirrors the practical assessment of pseudothrombocytopenia through analyzer flags, platelet histograms, channel discordance, smear morphology, sample timing, and clinical context. Literature on responsible clinical AI implementation highlights the importance of transparency, documentation, professional accountability, privacy safeguards, and human oversight [20], all of which are important when an algorithm may influence whether a platelet count is released, withheld, qualified with an interpretive comment, repeated, or escalated for expert review. Clinical AI studies using causal machine learning and stratified causal inference illustrate methods for examining heterogeneous effects, subgroup-specific patterns, and outcome-oriented prediction [21,22]; similar study designs could help determine whether AI-triggered reflex pathways affect repeat sampling, smear review, transfusion avoidance, hematology referral, procedural delay, or other downstream decisions across different patient groups and laboratory settings. Work on precision-recall trade-offs in surgical AI highlights that model thresholds should be interpreted in relation to the consequences of false-positive and false-negative outputs [23], a key issue for pseudothrombocytopenia workflows in which missed platelet clumps and unnecessary smear reflexes carry different clinical and operational risks. TabPFN-based precision modelling in surgical health economics provides an example of evaluating machine-learning systems beyond technical accuracy, including cost, workflow, and real-time decision support [24]; future pseudothrombocytopenia studies should similarly consider repeat testing, staff time, turnaround time, avoided interventions, and clinician callbacks. Finally, reports on AI readiness among health professionals [25] and medical students [26] indicate that implementation depends on whether end users are prepared to interpret, question, and override AI-supported outputs when required. Together, these considerations provide a framework for future pseudothrombocytopenia-specific validation studies that assess not only diagnostic performance, but also safety, workflow value, cost, and clinical impact.

These future research priorities converge on a practical model of multidisciplinary laboratory stewardship for AI-supported pseudothrombocytopenia workflows. In this model, AI would not function as an independent diagnostic authority. Instead, it would assist trained laboratory professionals by integrating full blood count parameters, platelet channel discordance, analyzer flags, sample timing, and digital smear findings; triggering locally validated reflex pathways; presenting visual evidence of possible platelet aggregates; and supporting clear interpretive comments when platelet counts are unreliable. The final decision, including whether to release, withhold, qualify, repeat, or escalate a platelet result, should remain with appropriately trained laboratory staff. Such an approach preserves the efficiency and standardisation offered by automation while maintaining expert accountability, reducing automation bias, and protecting patient safety.

## Conclusions

Pseudothrombocytopenia detection can benefit from AI when the technology is embedded within a defined hematology workflow. The optimal model integrates analyzer flags, alternative platelet channels, digital morphology, AI-supported triage, clear reporting, and expert review. The current evidence supports feasibility and workflow value, but it does not justify autonomous AI diagnosis or unvalidated cross-platform adoption. Further research should focus on external validation, direct comparison with conventional workflows, false-positive and false-negative consequences, cost-effectiveness, and patient-centred outcomes. AI should therefore augment, not replace, expert interpretation in the detection and reporting of artefactually low platelet counts.
